# Continuous Wave Fractional CO_2_ Laser for the Treatment of Upper Eyelid Dermatochalasis and Periorbital Rejuvenation

**DOI:** 10.1089/pho.2016.4225

**Published:** 2017-05-01

**Authors:** Melissa Morrison Toyos

**Affiliations:** Toyos Clinic, Nashville, Tennessee.

**Keywords:** aging, blepharoplasty, continuous wave CO_2_ laser, dermatochalasis, fractional CO_2_ laser, periorbital rejuvenation

## Abstract

Fractional continuous wave CO_2_ laser resurfacing is used to improve photodamage, wrinkles, and acne scarring by surface ablation and by using heat to activate natural collagen production and dermal remodeling. In this study, the author examined the efficacy and safety of nonincisional continuous wave fractional CO_2_ laser blepharoplasty in the upper lid. Standard lid measurements including marginal reflex distance, palpebral fissure, and upper lid crease were performed preoperatively and at 6 months by the surgeon. All patients underwent full facial MIXTO continuous wave CO_2_ laser treatment (MIXTO Slim Evolution 2; MIXTOLasering USA, San Ramon, CA), including resurfacing on the upper eyelid from lashes to brow. We evaluated results at 6 months after laser treatment and found that on average, after MIXTO continuous wave laser treatment, marginal reflex distance of the upper lid increased from baseline from 0.7 to 2.2 mm, palpebral fissures increased from 5.6 to 7.4 mm, the upper lid crease was unchanged at 5.7 mm as was the upper lid excursion at 14.7 mm compared with those before treatment. Patients reported postoperative erythema, edema, crusting, and oozing that resolved within 14 days. These data demonstrate the safety and efficacy of noninvasive continuous wave fractional CO_2_ laser in the treatment of mild and moderate upper eyelid dermatochalasis.

## Introduction

Aging of skin is a natural, multifactorial process that results in skin thinning, skin laxity, wrinkles, and gravitational changes. Extent of aging is determined by internal factors related to genetics, skin pigmentation, and skin thickness as well as external factors such as sun exposure, smoking, environmental, and nutritional status. As the focal point of the face, aging can affect the periorbital region in significant ways even in young patients. Blepharoplasty is one of the most commonly performed procedures in aesthetic facial surgery.^1^ More than 165,000 procedures were performed in America in 2014 according to the American Society for Aesthetic Plastic Surgery.^2^ Upper eyelid thinning, hooding, and drooping of the upper lid skin are common. Topical therapies to enhance collagen production and reverse aging such as tretinoin (Retin-A), Vitamin C, and alpha and beta hydroxyacids can be effective but have limitations.^3,4^ Surgical revision of dermatochalasis is the gold standard for treatment, but can expose patients to pain, bruising, and prolonged recovery times.^5^ Fractional continuous wave CO_2_ laser generates more residual heat in skin and induces immediate tissue tightening through selective skin vaporization collateral thermal effects and long-term collagen stimulation by stimulation of fibroblasts from the enhanced dermal heat.^6,7^ Laser rejuvenation of upper lid skin using continuous wave CO_2_ laser is low cost, noninvasive, has shorter operative and recovery times with more natural outcomes, and no scarring compared with traditional surgical techniques.^8,9^ The aim of this study was to test the hypothesis that noninvasive fractional CO_2_ laser could be safely used as an alternative method of periorbital rejuvenation.

## Methods

In this retrospective study, we reviewed charts and photographs of patients undergoing full face continuous wave laser facial resurfacing, including upper lid resurfacing and performed at baseline and 6-month evaluations. All patients signed an informed consent. The study included 14 female Caucasian patients, 1 female Pacific Islander, and 1 female Asian patient. Mean age was 53 years of age (range 34–68). Patients were recruited from May of 2013 to August of 2015 and the study was completed in November of 2015. The inclusion criteria were skin phototypes I–IV and presence of dermatochalasis.

Exclusion criteria included active infection, compromised immune function, history of keloids, botulinum injection within 6 months, history of filler injection in the periorbital area, prior upper lid laser, prior surgical procedures in the upper lid, photosensitivity, pregnancy, lactation, oral retinoid in the past year, eyelid malposition (entropion, ectropion, blepharoptosis, and retraction), abnormal eyelid movements (blepharospasm and seventh nerve palsy), significant brow ptosis as determined by the surgeon, and patients not considered to be able to follow treatment protocol.

All subjects completed a baseline clinical examination, including slit lamp, fundus, and lid examination, including measurement of marginal reflex distance, upper lid crease distance, upper lid excursion, and palpebral fissure distance. Photographs were taken in frontal and lateral projections using Panasonic GH3 Lumix digital camera. Photographs were standardized in magnification, lighting, and positioning. Photographs were excluded if patients had partially closed lids.

All subjects included in the study returned for 6-month evaluation, which included slit lamp examination and lid measurements of marginal reflex distance, upper lid crease distance, upper lid excursion, and palpebral fissure distance. Photographs were repeated in the frontal and lateral projections using the same camera, standardized in magnification, lighting, and positioning. Photographs were again excluded if patients had partially closed lids.

### Laser technique

Local cutaneous anesthesia (lidocaine 23% and tetracaine 7%) was applied in a thin film two times 45 min apart before the procedure. Oral oxycodone with ibuprofen was given as well as oral promethazine and alprazolam. Honeywell IPL-Aid topical lid stickers were applied for ocular protection. The patient was placed in the supine position with the operator behind the patient's head. The facial and supraorbital regions were treated from the upper lid lashline to just under the brow with the 300 and 180 μm handpieces in two passes each, titrating the laser power to observable skin retraction. The passes were made at a 45° angle to each other. Laser settings were 20% density, indexes of six or eight depending on skin type, and power of 10–15 W depending on skin type and skin reaction. All procedures were completed using the MIXTO fractional CO_2_ laser (MIXTO Pro Slim Evolution II). Immediate postoperative care included a normal saline rinse of the skin, a topical vitamin C cream, and Vaniply, a fragrance-free noncomedogenic ointment (PSICO, Rochester, MN).

## Results

A total of 16 patients with mild to moderate upper eyelid dermatochalasis were treated using fractional continuous wave CO_2_ laser. Patients were seen for evaluation of final clinical outcomes at 6 months after a single laser treatment. Patients reported minimal discomfort during the procedure and minor discomfort afterward, controlled with oral nonsteroidals. Side effects were mild, with subjects reporting crusting, oozing, and edema that resolved within 10 days.

**FIG. 1.**
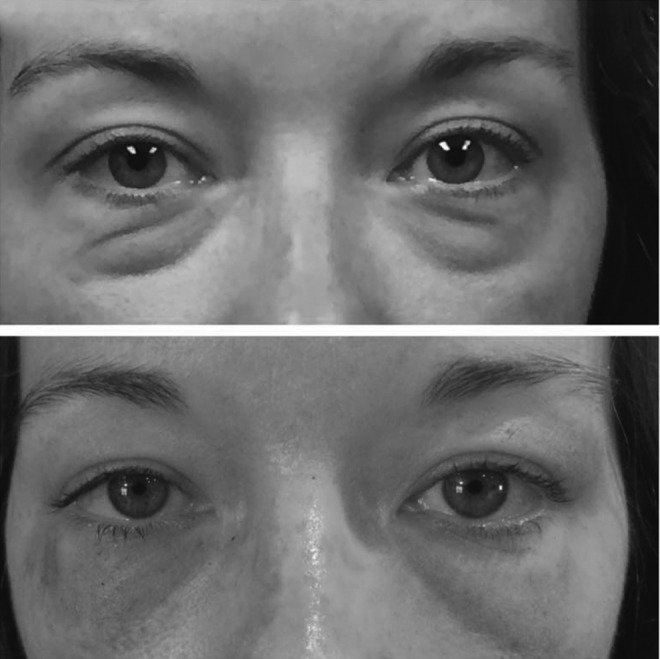
Baseline and immediate posttreatment photo of lower lids. (Patient consent obtained.)

**FIG. 2.**
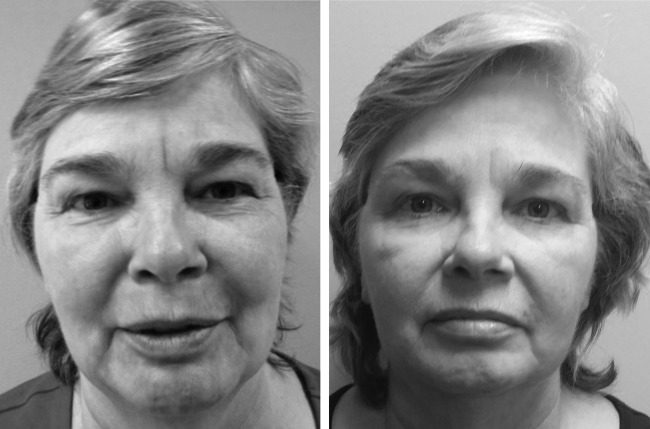
Baseline and 1-month postoperative appearance of upper and lower lids. (Patient consent obtained.)

**FIG. 3.**
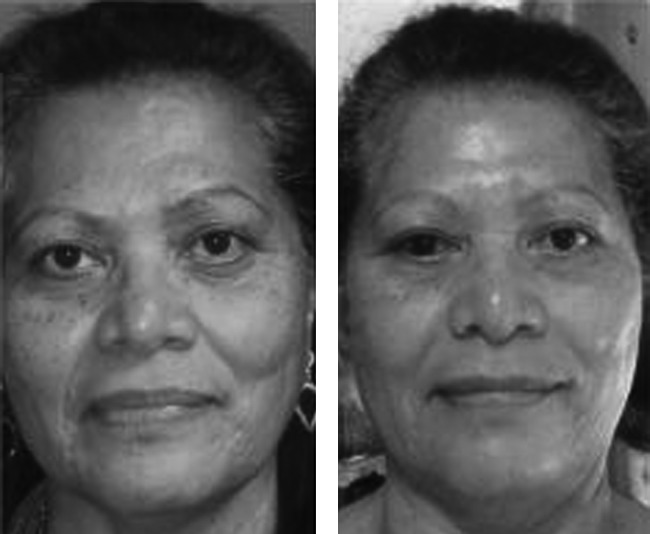
Baseline and 3-month postoperative appearance of upper and lower lids. (Patient consent obtained.)

We measured the results in terms of the change in elevation of the upper eyelid marginal reflex distance, the upper lid crease, upper eyelid crease position, and palpebral lid fissure distance from baseline. Six months after one MIXTO CO_2_ continuous wave laser treatment, the marginal reflex distance of the upper lid was increased on average from 0.7 to 2.2 mm (*p* = 0.09), palpebral fissure distance increased from 5.6 to 7.4 mm (*p* = 0.2), and upper lid crease was stable and unchanged at 5.7 mm (*p* = 0.07) as compared with that before treatment and upper lid excursion was unchanged at 14.7 mm (*p* = 0.1) (Figs. 1–3, Table 1).

**Table 1.** Lid Parameters Before and After Treatment

*Lid measurement*	*Baseline (mm)*	*6 months postoperative (mm)*
Margin reflex distance	0.7	2.2
Palpebral fissure	5.2	6.7
Upper lid crease	5.5	5.3

## Discussion

Dermatochalasis is a common side effect of aging that can be functional as well as cosmetic.^10^ As the focal point of the face, rejuvenation of the periorbital area presents unique challenges related to the function and safety of the eye.^11^ Noninvasive but effective laser procedures that reduce pain, swelling, downtime, and scarring would offer a unique benefit to patients seeking lid rejuvenation.

Traditional surgical blepharoplasty is currently the gold standard in periorbital rejuvenation but commonly causes pain, bruising, edema, and the potential for serious complications. The postoperative healing period is typically 6 weeks.^12^ Scarring is a feature of all incisional surgical procedures and can also be cosmetically undesirable.

There are many limitations to this study. Although it is prospective, it is not randomized and all patients received laser treatment as opposed to receiving no treatment or traditional surgical blepharoplasty. There is regional bias in that all patients were from a common geographic territory, all were female with most being Caucasian. All were treated by one surgeon in one center. In addition, the number of patients treated is small and the study is not powered to show strong statistical differences.

In general, CO_2_ lasers including fractional use can cause serious side effects such as hyper- or hypopigmentation, recurrence of viral illness, scarring, and infection. When used near the eye, reported side effects include corneal or scleral burns, loss of vision, and ectropion. Newer techniques leave a healthy rim of tissue surrounding the treated area and utilize a nonsequential microspot to minimize collateral thermal damage. These advances minimize, but do not eliminate, pain and side effects associated with CO_2_ laser.

Newer version of CO_2_ lasers maximizes thermal effect but minimizes the buildup of heat, reducing pain, side effects, and downtime. Continuous wave lasers are unique in that they use lower laser energies to build heat in target tissues over longer periods of time. Continuous wave lasers are currently popular in other areas of ophthalmology, primarily glaucoma and retina. The micropulse laser trabeculoplasty (Iridex, Mountain View, CA) lowers intraocular pressure similarly to the selective laser trabeculoplasty (SLT; Lumenis, San Jose, CA) but with less inflammation and no structural damage to the trabecular meshwork by ultrasound microscopy compared with SLT.^13^ The continuous wave retinal diode laser (Iridex) is the first of its type to be able to eliminate macular edema and drusen without scarring or damaging delicate retinal tissue.^14^

The MIXTO laser uses a 10,600 nm wavelength and a continuous wave micropulsed laser to separate microbeam impulses by time and space to reduce thermal injury, allowing the treated area to cool between pulses. The microspot reduces the time to reepithelialization from weeks to 1–2 days, reducing infection risk because of delayed epithelialization.^15^

## Conclusions

Surgical or invasive blepharoplasty has long been the gold standard for rejuvenation of upper eyelid dermatochalasis, although patients routinely experience bleeding, bruising, edema, scarring, and downtime as well as the risks of serious complications such as retrobulbar hemorrhage and dryness, causing temporary or permanent loss of vision. Continuous wave microspot lasers are known to generate greater amounts of nonscarring heat within skin than other CO_2_ lasers currently on the market to stimulate skin to naturally tighten both immediately and long term and improve texture and resistance to gravitational effects. Procedures can be performed in the office and outside of traditional operating rooms, reducing costs to both patients and practitioners. This laser provides decreased recovery period, higher safety profile, and the potential for more natural cosmetic outcome compared with traditional blepharoplasty.

This study evaluated improvements in common lid measurements for dermatochalasis and skin laxity by using a fractionated continuous wave CO_2_ laser in patients with dermatochalasis over a 6-month period after laser resurfacing of the upper lid area.
